# HTLV-I Tax Increases Genetic Instability by Inducing DNA Double Strand Breaks during DNA Replication and Switching Repair to NHEJ

**DOI:** 10.1371/journal.pone.0042226

**Published:** 2012-08-20

**Authors:** Hicham H. Baydoun, Xue Tao Bai, Shary Shelton, Christophe Nicot

**Affiliations:** University of Kansas Medical Center, Department of Pathology and Laboratory Medicine, Kansas City, Kansas, United States of America; Johns Hopkins School of Medicine, United States of America

## Abstract

**Background:**

Appropriate responses to damaged DNA are indispensible for preserving genome stability and preventing cancer. Tumor viruses often target DNA repair machinery to achieve transformation. The Human T-cell leukemia virus type I (HTLV-I) is the only known transforming human retrovirus and the etiological agent of Adult T-cell Leukemia (ATLL). Although HTLV-I-transformed leukemic cells have numerous genetic lesions, the precise role of the viral *tax* gene in this process is not fully understood.

**Results:**

Our results show a novel function of HTLV-I oncoprotein Tax as an inducer of genomic DNA double strand breaks (DDSB) during DNA replication. We also found that Tax acts as a potent inhibitor of homologous recombination (HR) DNA repair through the activation of the NF-kB pathway. These results were confirmed using HTLV-I molecular clones expressing Tax at physiological levels in a natural context. We further found that HTLV-I- and Tax-transformed cells are not more susceptible to DNA damaging agents and repair DNA lesions at a rate similar to that of normal cells. Finally, we demonstrated that during S phase, Tax-associated DDSB are preferentially repaired using the error-prone non-homologous end joining (NHEJ) pathway.

**Conclusions:**

This study provides new insights in Tax effects on DNA repair and genome instability. Although it may not be self sufficient, the creation of DNA breaks and subsequent abnormal use of the non-conservative NHEJ DNA repair during the S phase in HTLV-I-infected Tax-expressing cells may cooperate with other factors to increase genetic and genome instability and favor transformation.

## Introduction

HTLV-I infects more than 25 million people world-wide and a significant percentage of infected individuals develop adult T-cell leukemia (ATLL) or HTLV-I-associated myelopathy (HAM/TSP) [1]–[5]. HTLV-I-associated diseases are fatal with limited therapeutic options. The mechanisms used by HTLV-I to transform human T-cells are still poorly understood. Unlike animal-transforming retroviruses, HTLV-I does not use proviral integration to activate an oncogene or inactivate tumor suppressor genes, and HTLV-I does not transduce an oncogene. Although Tax has weak oncogenic activity in human T-cells, the genomic and genetic instability caused by the viral Tax is thought to play an important role in ATLL development [6]–[8]. Tax transforms murine fibroblasts in vitro and is associated with the development of various tumors in vivo in transgenic models. The mechanisms used by Tax to transform cells are not clearly understood. Tax has been shown to constitutively activate NF-kB [9]–[12] and to stimulate cell proliferation [13]–[22], and both events seem to be required for Tax-transforming activities. Tax has been shown to inactivate key tumor suppressors, including p53. Tax also inhibits apoptosis pathways and activates hTERT, thereby extending the lifespan of infected cells. Finally, Tax prematurely activates the anaphase promoting complex [23]–[26], inhibits nucleotide excision repair [27]–[29] and alters topoisomerases [30], [31] and beta-polymerases [32] leading to increased genomic and genetic instability. Recently Tax has also been shown to associate with the mini-chromosome maintenance MCM2-7 helicase and stimulate S phase progression but also generates a genomic lesions [33]. Our data demonstrate that Tax induces DNA double strand breaks (DDSB) and inhibits DNA repair through the homologous recombination (HR) pathway. In addition, we showed that DDSB are repaired through the error-prone non-homologous end-joining (NHEJ) pathway. Since Tax is known to induce both genetic and chromosomal instability, understanding how Tax affects these pathways is essential for understanding HTLV-I-associated leukemia.

## Materials and Methods

### Cell lines

HTLV-I-transformed Cell lines MT-2, MT-4 and C8166 [34] were cultivated in RPMI 1640 (Gibco) with 10% fetal bovine serum (Gibco), supplemented with 2 mM glutamine, 1% penicillin-streptomycin and 0.4% gentamicin. Cell lines immortalized by HTLV-I, such as 1185, LAF, or that immortalized by Tax, such as WT4, WT4B and WT4I [35], were cultivated in the presence of IL-2 (50 U/ml, Roche Molecular).

### Cell cycle and Flow Cytometry analyses

For cell cycle synchronization and release, cells were treated overnight with Hydroxyurea (2 mM) to arrest cells in the G1 phase of the cell cycle. For the cell cycle distribution analysis, cells were resuspended in media containing the Dye Cycle Violet (Excitation at 405 nm and Emission at 450 nm) (Invitrogen) and incubated for 30 min at 37°C before being analyzed by an LSRII flow cytometer.

### Immunofluorescence and Microscopy

Cells were centrifuged on slides at 800 rpm for 5 min. They were then fixed in 3.7% paraformaldehyde (PFA) for 15 min at RT, washed with PBS, permeabilized on ice for 5 min with 0.5% Triton X-100 and blocked for 1 h in PBS with 0.5% gelatin and 0.25% bovine serum albumin at room temperature. For γ-H2AX staining, slides were incubated with anti γ-H2AX polyclonal antibody (Cell Signaling #2577) 1/200 in PBS for 2 h, washed three times in PBS-0.2% gelatin for 10 min each time, and incubated with Alexa Fluor 488-conjugated goat anti-rabbit secondary antibody (Molecular probes, Invitrogen) in PBS-0.2% gelatin for 1 h at room temperature. Cells were washed three times in PBS-0.2% gelatin for 10 min each time and mounted by using DABCO mounting medium (2.5% DABCO from Sigma, 200 mM Tris-HCl pH 8.6 and 90% glycerol). The same procedure was performed for dual staining of γ-H2AX and RAD51 (Abcam (#46981)) or γ-H2AX and Ku80 (Cell Signaling (#2753)). Fluorescent images were captured by using a Nikon TE2000E epifluorescence microscope and the Metamorph software (Molecular Devices). Optical sections through the nuclei were captured at 0.5-µm intervals, and the images were obtained by stitching of the individual sections. For quantitative analysis, foci were counted by manual counting on the collected images by using the 100× objective.

### Comet assay

Comet assay was performed using the Trevigen kit (#4250-050-K) according to manufacturer's instructions. We used the Neutral Comet Assay to detect double-stranded breaks specifically, whereas the Alkaline Comet Assay is more sensitive but detects both single and double-stranded breaks.

### BrdU incorporation assay

HTLV-I-transformed MT-2 cells were incubated with 100 uM BrdU (Sigma) for 1 h and were immediately fixed in 3.7% PFA for 15 min. Cells were first stained with γ-H2AX (primary and secondary antibodies as described above) and then fixed again in 3.7% PFA for 15 min to crosslink the pre-established bonds. Cells were subsequently treated with 2 N HCl for 30 min at 37°C followed by wash 2× for 5 min each with 0.1 M Borate Buffer pH 8.5 and then processed for immunofluorescence staining with anti BrdU antibody (Invitrogen).

### Western Blot

Cell extracts were prepared using NP-40 lysis buffer (1% NP-40, 50 mM Tris-HCl (pH 7.5)), 150 mM NaCl, and protease inhibitors. Proteins were separated by SDS page, transferred to PVDF membrane and western blotted using actin (Santa Cruz), Tax mouse monoclonal (NIH AIDS Reagent Program, HTLV-I Tax Hybridoma (168B17)) or anti γ-H2AX polyclonal antibody. Signals were revealed with super signal chemiluminescent (Thermo Scientific).

### Amaxa transfection

Jurkat cells were transfected using the Nucleofector Kit #V (Amaxa Biosystems) according to manufacturer's instructions. 48 hours after transfection cells were collected washed in PBS and analyzed.

### Lentivirus preparation and transduction of Jurkat cells

Semi-confluent 293T cells plated in 150 mm were transfected with packaging plasmid pDNL6 (10 µg), pHRCMVTax (20 µg) or pHRCMVGFP, and pVSV-G (10 µg) using calcium phosphate (Invitrogen) according to the manufacturer's instructions. Supernatant was collected at 24, 48 and 72 hours and virus particles concentrated by ultracentrifugation. Concentrated virus was used to infect Jurkat cells in the presence of polybrene 5 µg/ml and cells were analyzed 48 hours later.

## Results and Discussion

### HTLV-I- and Tax-expressing cells have a high rate of DNA double strand breaks (DDSB) in the absence of exogenous treatment

**Figure 1:**
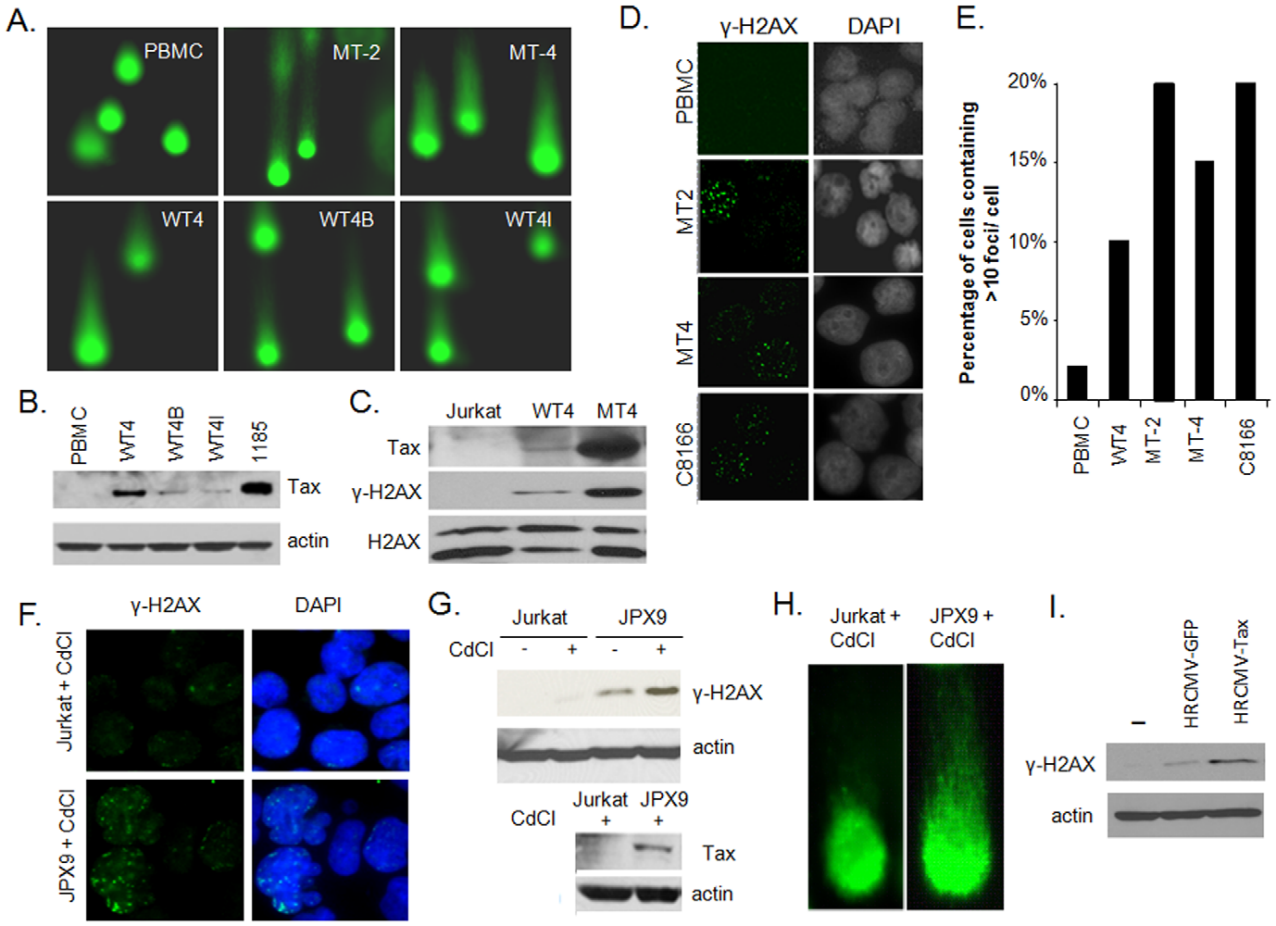
HTLV-I Tax stimulates the formation of DDSB in the genome. A) Accumulation of DDSB in HTLV-I-transformed cells MT-4 and Tax-only immortalized human T cells WT4, WT4I and WT4B was analyzed by comet assays. Normal PBMCs were used as a negative control. B) Western blots analyses showing physiological levels of Tax expression in human T-cell lines immortalized by Tax only. C) Western blot analyses for γ-H2AX and Tax expression in HTLV-I-transformed MT-4 cells, Tax-immortalized WT-4 T-cells or Jurkat control cells. H2AX was used as a loading control for each sample. D) γ-H2AX foci were readily detected by immunofluorescence in HTLV-I transformed cells and Tax-expressing T-cells but not in normal PBMCs. E) Quantification of the percentage of cells harboring 10 or more γ-H2AX foci per cell in PBMC, WT-4, MT-4, MT-2 and C8166. Results were derived from counting 100 nuclei for PBMC and each cell line. F) Tax-inducible Jurkat JPX9 cells and Jurkat control cells were treated with CdCl to induce Tax expression in the former and analyzed by immunofluorescence for presence of DDSB as shown by γ-H2AX foci. G) Western blot analyses using γ-H2AX and Tax-specific antibodies confirmed an increased γ-H2AX expression in Tax-expressing cells only. H) Representative image of DDSB detected by comet assays in JPX9 cells only after induction of Tax and not Jurkat cells treated under the same conditions. I) Jurkat cells were transduced with VSV-pseudotype viruses expressing Tax or GFP as described in materials and methods. After 48 hours proteins were extracted and analyzed by western blot for γ-H2AX expression. Actin was used as a loading control.

The unusually high basal level of phosphorylated ATM (S1981) in HTLV-I-transformed cells [36] suggests an accumulation of DDSB, the most serious form of DNA damage, because they pose problems for transcription, replication, and chromosome segregation [37]. We previously showed that immortalization of human primary T cells by Tax alone is an infrequent and long process, suggesting that it requires additional genetic events [35] and possibly additional viral genes for efficient immortalization/transformation. Tax-immortalized human T cell line WT4 and two subclones, WT4I and WT4B, were analyzed by single cell gel electrophoresis assay or comet assay, a sensitive method for the detection of DNA strand break damage in cells. In this assay, the tail represents damaged DNA, and the brighter and longer the tail, the higher the level of damage. Together our data demonstrate that HTLV-I- and Tax-expressing cells have a high rate of DDSB in the absence of any external treatment when compared to normal PBMCs (Fig. 1A). Importantly, these effects were not mediated by over-expression of Tax since Tax-immortalized T-cell lines express Tax at physiological levels comparable to 1185, an HTLV-I-immortalized cell line (Fig. 1B). To further investigate the presence of DDSB in HTLV-I-transformed cells, levels of H2AX and phosphorylated-H2AX (referred to as γ-H2AX) were analyzed in HTLV-I-transformed MT-4 cells and Tax-expressing T-cells WT-4 and control PBMC (Fig. 1C). Results indicated a significant increase in γ-H2AX suggesting a steady state level of DNA damage in both MT-4 and WT-4. Levels of γ-H2AX also appear to correlate with the lower levels of Tax expression in WT-4 cells. Although we cannot exclude that additional viral proteins such as p30 or HBZ expressed in MT-4 may contribute to higher γ-H2AX, our results clearly demonstrate that Tax alone (as in WT-4) is sufficient to induce γ-H2AX (Fig. 1C–E). To further confirm these data, we immune-stained HTLV-I-transformed and Tax-expressing T-cell lines with an antibody specific for γ-H2AX. Our data confirmed the presence of numerous DDSB in HTLV-I- and Tax-expressing T-cell lines when compared to normal PBMCs (Fig. 1D). We ensure the statistical significance of these data as the number of foci per cell was counted in 100 nuclei for each cell line. Results represent the percentage of cells containing 10 or more γ-H2AX foci per cell (Fig. 1E)

Since the DDSB seen in HTLV-I- or Tax-expressing cell lines could be due to adaptation and/or genetic alteration rather than Tax expression itself, we used a stable Jurkat T-cell line carrying the *tax* gene under the control of an inducible promoter (JPX9) [38]. Tax expression is induced at physiological levels when these cells are exposed to CdCl. Importantly, induction of Tax expression in JPX9 cells was associated with a significant increase in γ-H2AX (Fig. 1F). The variable number of γ-H2AX foci observed in JPX9 cells induced with CdCl was presumably because not every cell has the same response to CdCl and Tax levels. However, the presence of DDSB was clearly specific to Tax expression since it was not observed in Jurkat control cells treated in the same conditions with CdCl (Fig. 1G). These data demonstrate that Tax induces either directly or indirectly DDSB in human T-cells. We also confirmed these results by Western blot analyses showing an increased expression of γ-H2AX in JPX9 cells, but not Jurkat exposed to CdCl (Fig. 1G) and comet assays (Fig. 1H). Finally, these results were confirmed in transient assays by transduction of Jurkat cells with high titer Tax-expressing pseudotype virus particles. As a control, Jurkat cells were infected with GFP-expressing lentiviral pseudotype virus particles. After 48 hours western blot analyses revealed a significant increase in γ-H2AX expression in Tax-transduced cells when compared with un-transduced or GFP-transduced Jurkat cells (Fig. 1I). All together, our results clearly demonstrate that Tax expression itself is sufficient and responsible for initiation of DDSB.

### Tax expression is associated with the formation of DDSB during DNA replication in S phase

**Figure 2:**
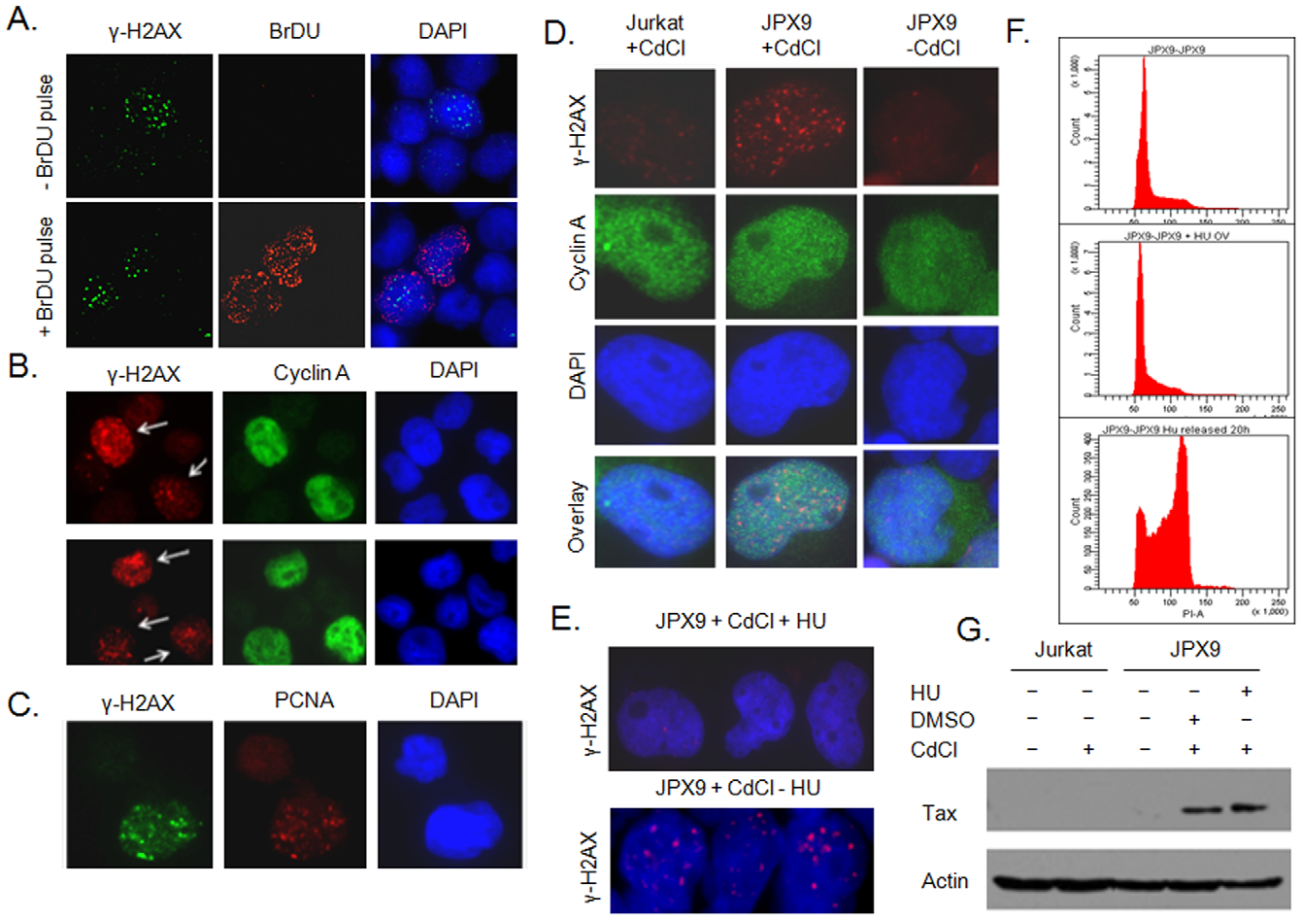
Tax-associated DDSB occur in S phase during DNA replication. A) HTLV-I-transformed MT-2 cells were pulse-labeled with BrDU to identify cells in S phase of the cell cycle. Immunofluorescence experiments indicated that γ-H2AX foci are detected in BrDU positive cells only. Cells were counter stained with DAPI. B) HTLV-I-transformed MT-2 cells were dual stained with Cyclin A, a marker of S phase, and γ-H2AX, a marker of DDSB foci. Cells were counter-stained with DAPI. C) HTLV-I-transformed MT-2 cells were dual stained with PCNA, which appear as a punctuated pattern in S phase, and γ-H2AX, to reveal DDSB foci. Cells were counter-stained with DAPI. D) Accumulation of DDSB foci in S phase was confirmed in Tax-only expressing cells using Tax-inducible JPX9 cells and Jurkat control, both treated with CdCl and dual-stained with Cyclin A and γ-H2AX antibodies. E) JPX9 cells were synchronized in G0/G1 by exposure to hydroxyurea (HU) overnight. G0/G1 JPX9 cells and non-synchronized JPX9 cells were exposed to CdCl to induce Tax expression and analyzed for presence of γ-H2AX DDSB foci. F) Efficacy of HU treatment was demonstrated by cell cycle analyses by FACS after PI staining. From top to bottom, untreated cells, HU G1-arrested cells, HU G1-arrested cells released by washing out HU and culturing 20 hours. G) Western blot analyses confirmed that HU treatment did not affect Tax expression.

We previously reported that HTLV-I p30 inhibits accurate DDSB repair occurring in S phase during DNA replication. Since Tax has been shown to affect cell cycle regulators, DNA polymerase and topoisomerase, we next investigated whether Tax-induced DNA breaks occur throughout the cell cycle or during DNA replication in the S phase. To answer this question we performed dual staining for γ-H2AX, to mark DNA breaks, and a short pulse of BrDU incorporation, to label replicating DNA in S phase in HTLV-I-transformed MT-2 cells. Our results clearly demonstrated that γ-H2AX foci were detected only in cells with replicating DNA (Fig. 2A). These findings were further confirmed by staining for γ-H2AX and Cyclin A, a marker of cells in S phase [39]–[43] (Fig. 2B). Consistent with our results above, cells positive for γ-H2AX were also positive for Cyclin A (Fig. 2B). Although one study reported that Tax represses Cyclin A expression in two HTLV-I cell lines, C8166 and MT-4 [44], the MT-2 cell line was not tested in that study and additional studies found expression of Cyclin A in HTLV-I transformed C8166 cells [45]. Our data also show that in HTLV-I Tax-expressing MT-2 cells Cyclin A is readily detectable (Fig. 2B). Finally, a similar conclusion was also reached using γ-H2AX and PCNA (Fig. 2C), for which a punctuated signal is indicative of cells in S phase [43]. Our results suggest that the induction of DDSB by Tax is global rather than occurring at limited specific DNA sites, as suggested by the accumulation of numerous γ-H2AX foci throughout the nucleus in the presence of Tax. Finally, we confirmed that Tax-mediated DDSB occur in S phase since γ-H2AX and Cyclin A were detected in JPX-9 cells only after Tax induction (Fig. 2D). To further confirm that Tax-associated breaks occur in S phase, we synchronized JPX9 cells in G0/G1 by hydroxyurea treatment as previously reported [33]. Following synchronization induction of Tax was no longer associated with DDSB (Fig. 2E), another argument that Tax induces breaks mainly during DNA replication in cells in S phase. Synchronization by HU was confirmed by cell cycle analyses after propidium iodide staining (Fig. 2F) and Tax expression was not affected by treatment with HU (Fig. 2G).

### HTLV-I- and Tax-transformed cells are more susceptible to DNA damaging agent but exhibit a repair rate similar to normal PBMC

**Figure 3:**
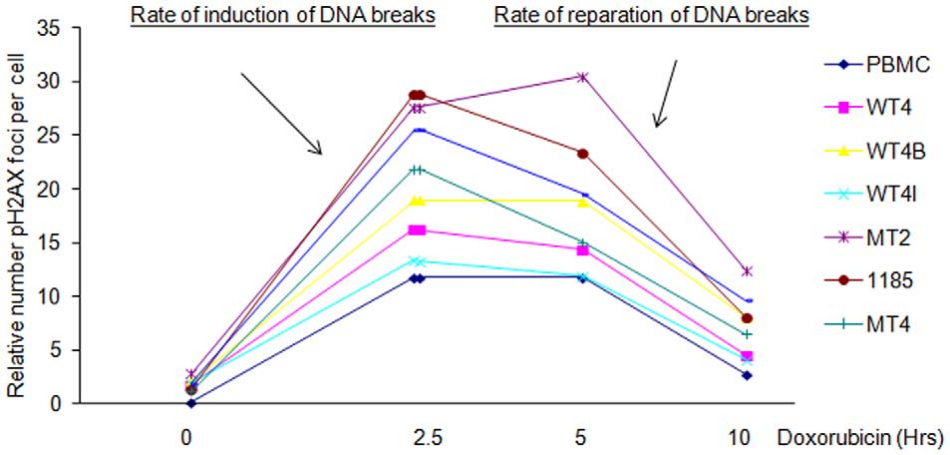
HTLV-I-transformed cells and normal PBMC are equally sensitive to DNA damaging agents and have an overall similar rate of DNA repair. HTLV-I-transformed cells (MT-2, MT-4, C8166, 1185) and Tax-immortalized cells (WT4, WT4B, WT4I) and PBMC were exposed to 10 nM of Doxorubicin, washed and rate of repair was evaluated by immunostaining of γ-H2AX -revealed DDSB foci and quantified by microscopy at 0 h, 2.5 h, 5 h and 10 h after treatment. Because of various numbers of breaks in the absence of treatment, DNA breaks were normalized at 0 for time T = 0. Data were generated from counting 100 cells. Rate of repair is represented by the decreasing number of γ-H2AX foci at 2.5, 5 and 10 hours after doxorubicin treatment. Data were generated from counting 100 cells.

Because Tax expression was associated with increased γ-H2AX foci formation these cells could be more sensitive to DDSB or have a repair rate slower than normal cells or both. To answer these questions we next investigated whether HTLV-I- and Tax-transformed cells may be more prone to DNA damage following exposure to external agents. Several HTLV-I- and Tax-transformed cell lines and control PBMC were exposed to Doxorubicin, a topoisomerase II inhibitor that induces DNA double breaks. The rate of induction of DNA breaks for each cell line was evaluated, at time t = 0 and 2.5 hours after exposure to Doxorubicin, by counting the relative number of γ-H2AX foci per cell in 100 cells. As shown by our results, DDSB induction is higher in HTLV-I cell lines tested compared to Tax-expressing T-cells (WT4, WT4B, and WT4I) and control PBMC (Fig. 3). These data suggest that HTLV-I- and Tax-transformed cells are more sensitive than normal cells to external DNA damaging agents. HTLV-I cell lines demonstrated the highest number of γ-H2AX foci per cell. These data may be related to the higher Tax expression in these cells compared with Tax expressing T-cell lines or additional viral genes and this warrants further study. Nonetheless, all three Tax-expressing cell lines had a higher number of γ-H2AX foci per cell when compared to PBMC (Fig. 3), suggesting that Tax alone is sufficient to sensitize T-cells to DNA damaging agents. We next measured the DDSB repair rate following doxorubicin exposure by counting the number of γ-H2AX foci per cell at five and ten hours after treatment. These experiments suggested that the initial early repair rate, from 2 to 5 hours, is either similar to normal PBMC or accelerated in 1185, MT-4 and C8166 as shown by the inclination of the slope (Fig. 3). However, five hours and onwards after DDSB formation, the rate of repair is equivalent in HTLV-I-transformed and normal cells.

### Tax-mediated NF-kB activation inhibits the HR DNA repair pathway

**Figure 4:**
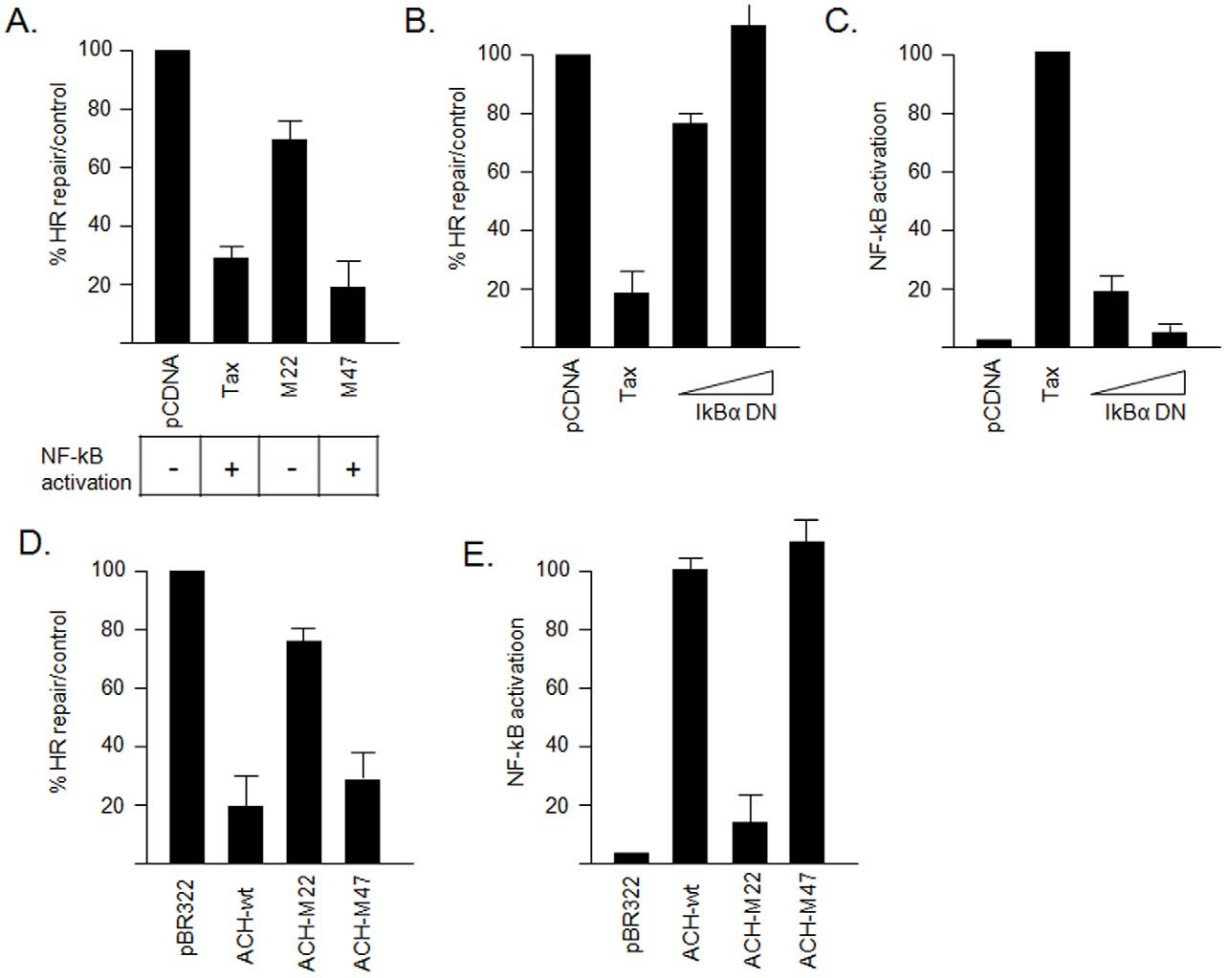
Tax inhibits the DDSB HR DNA repair through activation of NF-kB. A) Representation of in vivo HR assays using the DR-GFP HR reporter and the pI-SceI vectors. B) Jurkat T cells were cotransfected with DR-GFP vector either with the control vector or with the pI-SceI, and along with Tax or Tax mutants M47 or M22 using Amaxa electroporation. Forty-eight hours later the expression of GFP was assessed by FACS analysis. Relative percentage of GFP-expressing cells was represented by histograms corresponding to the average of 3 independent experiments. C) Activation of NF-kB reporter luciferase vector by HTLV-I Tax and Tax mutants M47 and M22. D and E) In vivo HR assay was performed in the presence of Tax and along with coexpression of a phosphorylation-defective dominant negative IkBα mutant that efficiently blocks NF-kB activation by Tax. F) Inhibition of HR by Tax was investigated using HTLV-I molecular clones expressing Tax or Tax mutants M47 and M22 at physiological levels.

DDSB generated during DNA replication are normally repaired by homologous recombination (HR) [46], [47]. Since Tax expression is associated with DNA breaks during S phase, we investigated the effect of Tax on HR DNA repair. To this end, we used an established *in vivo* Homologous Recombination Assay based on the DR-GFP HR reporter vector [48] and FACS analysis. The system utilizes a modified gene for GFP as a recombination reporter and expression of *Sce* I endonuclease for the creation of DDSB. Jurkat T cells were transfected by Amaxa with Tax and Tax mutants. Our results demonstrated that Tax expression results in 80% inhibition of HR DNA repair (Fig. 4A). To determine the signaling pathway responsible, we used a Tax mutant selectively defective for some of the wild type Tax activity. The Tax M22 mutant activates the cyclic AMP pathway (CREB/ATF) but not the NF-κB pathway [49]. On the other hand, Tax M47 activates the NF-κB pathway but not the CREB/ATF pathway [49]. Our experiments showed that Tax-mediated NF-kB activation is important for inhibition of HR DNA repair since both Tax and M47 significantly inhibited HR but not the M22 mutant (Fig. 4A). Previous studies have shown that M22 and M47 are expressed at comparable levels [50]. We repeated the assay using increasing amounts of IkBα-DN (SS32/36AA), a mutant that cannot be phosphorylated and blocks Tax-mediated NF-kB activation (Fig. 4C). We previously showed that IkBα-DN does not alter Tax expression, localization or transactivation functions [51]. Consistent with our hypothesis, IkBα-DN efficiently blocked Tax-mediated inhibition of HR activity (Fig. 4B). Together our findings highlight a novel role of Tax-mediated NF-kB activation in the inhibition of HR DNA repair.

Next, we used HTLV-I infectious molecular clones, ACH, and ACH-M22 or ACH-M47 [52], in which the *tax* gene was replaced with a *tax* mutant sequence in order to confirm the above results using a more physiological and regulated expression of Tax. Results presented in figure 4D confirmed that, in these experimental conditions, expression of both ACH and ACH-M47 in human Jurkat T cells inhibits HR DNA repair, while ACH-M22 had no significant effects. Proper activation of the NF-kB pathway by ACH-wt and ACH-M47 but not ACH-M22 was verified (Fig. 4E). All together, these findings confirm that Tax expression in a natural and physiological context and Tax-mediated NF-kB are critical for the inhibition of HR DNA repair.

### Single strand annealing (SSA) or microhomology-mediated end joining (MMEJ) are not frequently used for the repair of DDSB in Tax-expressing cells

**Figure 5:**
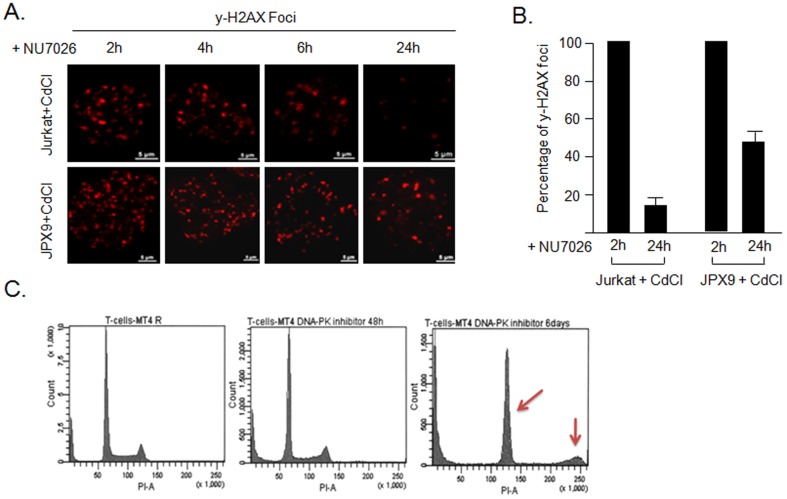
Tax-associated DDSB are not repaired by SSA or MMEJ. A and B) Tax-inducible cell line JPX9 and Jurkat control cells were induced with CdCl, γ-irradiated with a dose of 2Gy and treated with or without DNA-PK inhibitor NU7026. γH2AX foci were detected by microscopy at time t = 0, 2 h, 4 h, 6 h and 24 h. Image results provided are representative of the experiment performed in duplicate. Average number of γ-H2AX foci per cell was quantified by microscopy. The error bars in the figures represent the standard error of the mean (SEM) for 30 to 50 cells per sample. The images were obtained using epifluorescence Nikon Ti-s and have been treated with the deconvolution feature provided with the Nikon's NIS-Element software. C) HTLV-I Tax-expressing cells MT-4 were untreated (left) or incubated with DNA-PK inhibitor for 2 days (middle panel) or 6 days (inhibitor was replenished every 2 days). Cell cycle was analyzed by FACS after propidium iodide staining. Arrows indicate cell cycle arrest in G2/M and aneuploidy.

The canonical Ku80-dependent NHEJ pathway is usually the preferred method of repair while alternative NHEJ (alt-NHEJ) SSA and MMEJ pathways are used upon loss of Ku80 [53]. Alt-NHEJ are error-prone mechanisms of repair and result in deletion/duplication, mutations and frequent chromosome abnormalities, and may initiate the creation of oncogenes and cancer [54], [55]. In contrast to NHEJ, alt-NHEJ repair occurs independently from Ku80 and DNA-PK. We reasoned that if alt-NHEJ is actively involved in the repair of Tax-associated DDSB, then inhibition of DNA-PK, only involved in NHEJ, should not significantly affect the rate of repair (rate of disappearance of γ-H2AX foci) in Tax-expressing cells. On the other hand, if NHEJ is required, use of the DNA-PK inhibitor should lead to a significant decrease in repair efficiency. We used MT-4, WT4, Tax-induced JPX9 cells and Jurkat or Kit-225 [56] irradiated with a dose of 2Gy to induce DNA breaks. Cells were cultured in the presence or absence of a DNA-PK inhibitor (NU7026) [57], [58] and the average number of γ-H2AX foci per cell was quantified by microscopy. Because y-irradiation produces breaks that are mainly repaired through NHEJ and the DNA-PK inhibitor (NU7026) is specific for NHEJ, our results are measuring NHEJ and are not affected by residual HR, negligible in these conditions. Our results (Fig. 5A and 5B) showed a significant impairment in repair efficiency in Tax-expressing cells (50% unrepaired at 24 h) compared with controls (10%). This suggests that Tax-associated DDSB require DNA-PK for efficient repair and, therefore, that alt-NHEJ is not frequently used to repair breaks in Tax-expressing cells. Since Tax inhibits the HR pathway, we reasoned that if Tax-expressing cells accumulate breaks in S phase, these breaks must be repaired by using the conservative NHEJ pathway, then the use of a specific DNA-PK inhibitor should prevent DNA repair in these cells, lead to unrepaired accumulation of DDSB and cell cycle arrest. To test this hypothesis we used DNA-PK inhibitor NU7026 to prevent the use of the canonical NHEJ-dependent repair in Tax-expressing cells. Consistent with our hypothesis, these experiments showed an accumulation of cells in G2/M, a polyploid population and increased sub G0/G1 population, suggesting high cell death (Fig. 5C). These results are consistent with our previous findings that the mitotic spindle checkpoint is active in Tax-expressing cells, which arrest in G2/M following treatment with Taxol or Nocodazole [35].

### Tax-associated DDSB are repaired mainly through error-prone NHEJ pathway

**Figure 6:**
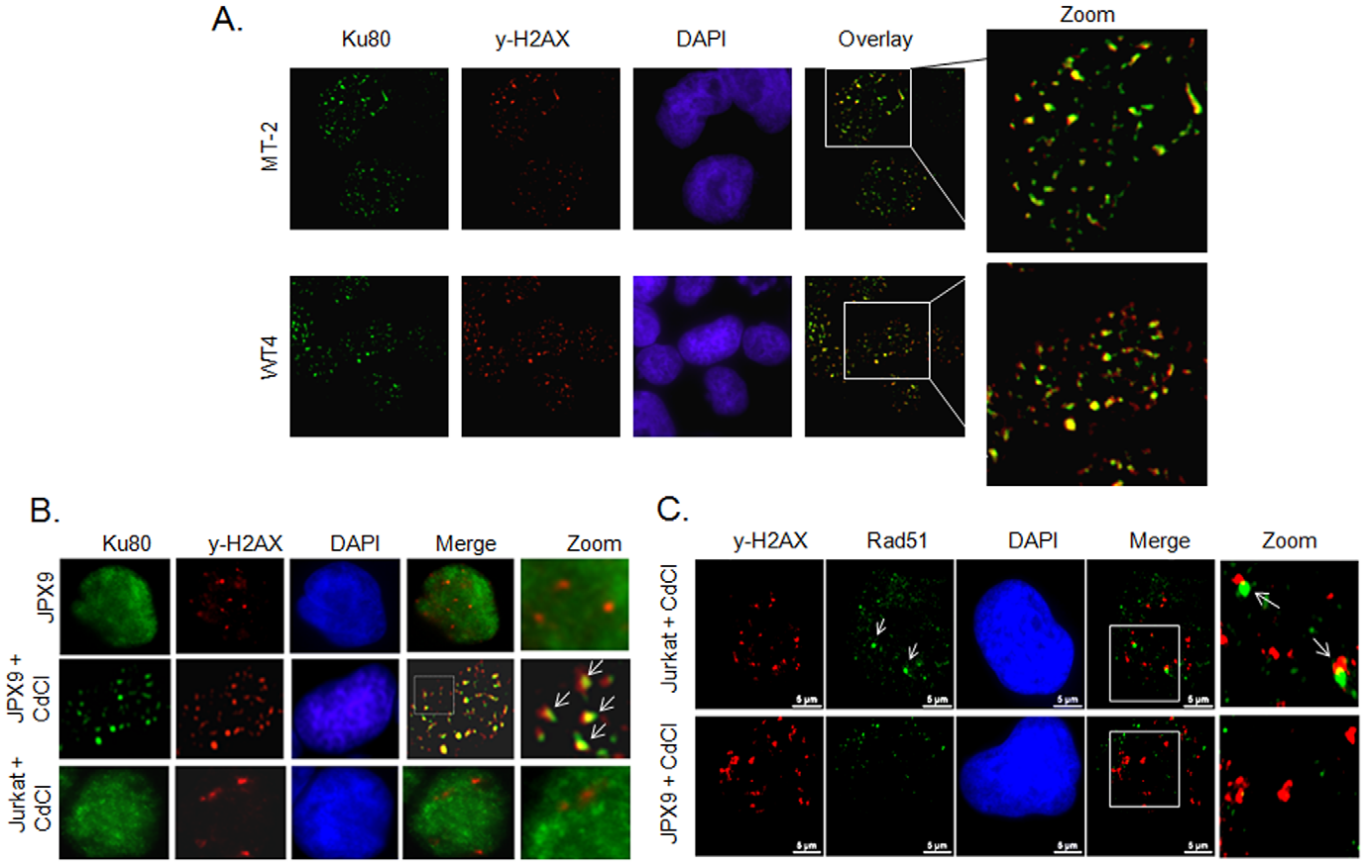
Tax expression switch DDSB repair from HR to error-prone NHEJ. A) Colocalization of Ku80 (specific to NHEJ DNA repair) and γ-H2AX in MT-2 and WT4 cells 30 min after gamma-irradiation. B) Colocalization of γH2AX foci and Ku80 in Tax-inducible JPX9 after induction of Tax expression by CdCl. Non-induced JPX9 and CdCl treated Jurkat control cells were used to demonstrate specificity to Tax expression. The images were obtained using epifluorescence Nikon Ti-s and have been treated with the deconvolution feature provided with Nikon's NIS-Elements software. C) Colocalization of γ-H2AX foci and Rad51 (specific to HR DNA repair) after Tax induction in JPX9 and in Jurkat cells that were used as control. The images were obtained using epifluorescence Nikon Ti-s and have been treated with the deconvolution feature provided with Nikon's NIS-Elements software.

Since Tax expression is associated with DDSB during S phase and Tax prevents their repair through HR, we expected an accumulation of Tax-expressing cells in the S phase. However, our data indicated no accumulation in the S phase of HTLV-I-infected cells or JPX9 cells after induction of Tax when compared to Jurkat control cells treated in the same conditions (data not shown). Similar results were also obtained after γ-irradiation or treatment with Aphidicolin (data not shown). Together these observations suggest that in Tax-expressing cells DDSB are repaired efficiently but mostly in an HR-independent manner. Repair through NHEJ depends upon Ku80 and DNA-PK recruitment to DDSB [59], [60] foci. Therefore, NHEJ-specific repair of DDSB can be detected by the colocalization of Ku80 and DNA-PK with γ-H2AX foci. To this end we used Tax-transformed-cells MT-2 and Tax-immortalized WT4, while transformed Jurkat and immortalized Kit225 human T-cell lines were used as negative controls. We found that both Ku80 and DNA-PK specifically colocalized with γ-H2AX foci on DNA breaks in Tax-expressing cells WT4 and MT-2 (Fig. 6A). Staining was specific for Tax-expressing cells and was not observed in negative Jurkat control cells or immortalized Kit225 human T-cell lines (not shown), Jurkat or uninduced JPX9 cells (Fig. 6B). To confirm a specific effect of Tax, we used Jurkat Tax-inducible JPX9 and Jurkat control cells. We found that a large number of γ-H2AX foci were colocalized with NHEJ-specific factor Ku80 after induction of Tax in JPX9 cells (Fig. 6B) as shown by yellow overlay and arrows. In contrast, Ku80 did not accumulate at all with γ-H2AX foci in uninduced JPX9 or Jurkat control cells (Fig. 6B). This data is consistent with previous reports showing that Ku80 staining is diffusely dispersed in the absence of NHEJ DNA repair (Fig. 6B). In contrast to NHEJ, repair through HR relies upon recruitment of Rad51 onto DDSB [61], [62]. Cells were treated with Aphidicolin to stimulate DNA replication-associated breaks. Under these experimental conditions Rad51 colocalized with γ-H2AX in Jurkat control cells but not in Tax-expressing cells (Fig. 6C). These results confirm that Tax expression reduces DDSB HR repair in favor of NHEJ-mediated repair.

## Conclusion

HTLV-I leukemic cells often present numerous genomic alterations, but the genesis and contribution of these chromosomal defects is unclear. The viral oncoprotein Tax has an important role in the initial stages of cellular transformation, inactivates tumor suppressors and stimulates cellular proliferation by inactivating several cell cycle checkpoints. In addition, several studies have shown that Tax inhibits multiple DNA repair pathways. We recently demonstrated that HTLV-I p30 disrupts the formation of the MRN complex onto DDSB foci induced during DNA replication in S phase. However, unlike Tax, our studies showed that p30 itself cannot induce DNA breaks directly, suggesting that it may cooperate with another viral gene to stimulate genetic instability. The data reported here indicate that Tax expression is sufficient to promote accumulation of DDSB in cells passing through the S phase. In this regard, it is important to note that Tax has been reported to inhibit PCNA, DNA Topoisomerase and DNA β-polymerase [63], [64], all known to be involved in DNA replication and to sequester DNA-PK, BRCA1, and MDC1 [65]. Previous studies have reported that Tax can inhibit p-ATM [66], although similar levels of p-ATM were observed 30 minutes after irradiation of control or HTLV-I cells. It is possible that 30 minutes is sufficient to allow activation of downstream targets and initiate DNA repair. Additional evidence of this study was derived from rat fibroblasts expressing Tax or high dose irradiated cells. Other studies found significant levels of p-ATM in HTLV-I-transformed MT-2 cells in the absence of irradiation or other treatment [36], which is consistent with results presented here and constitutive activation of the DNA repair response in Tax-expressing cells. Discrepancy between these studies may be related to the use of high dose irradiation or different lines being tested (MT-4 and Hut102 versus MT-2 and C10MJ). Our studies suggest that in HTLV-I infected cells Tax impairs HR and favors NHEJ DNA repair. An important component of the NHEJ pathway is Ku80. A previous study reported that Tax is associated with quantitative reduction of Ku80 expression but not depletion [67] and this explains why we were able to detect expression by immunofluorescence. Our data reveal a significant qualitative difference in Ku80 distribution within JPX9 cells following induction of Tax expression. In the absence of Tax, Ku80 appear diffuse, while in the presence of Tax and formation of DNA breaks, Ku80 accumulated at DNA break foci as previously reported [68].

Although our data suggest that Single strand annealing (SSA) or microhomology-mediated end joining (MMEJ) are not frequently used for the repair of DDSB in Tax-expressing cells, we cannot formally exclude the possibility that alt-NHEJ is being used at a lower frequency or specific breaks due to local chromatin structure, and additional studies will be needed to answer these questions. In contrast to HR and NHEJ, RAD52 and ERCC1 are important to promote SSA. Whether Tax affects RAD52 and/or ERCC1 remain to be demonstrated. How Tax-mediated NF-kB activation interferes with HR DNA repair is currently under investigation. An interesting possibility is the effect of Tax on p53. Independent studies showed that p53 can down-regulate HR activity and that this effect of p53 is independent from p53 transcription [69] but related to relative levels of expression, because mutations in the transcriptional domain of p53 do not affect inhibition of HR [70]. Interestingly, both Tax and Tax mutant M47 that inhibit HR activate NF-kB but also stabilize p53 protein levels. Whether Tax-mediated p53 protein stabilization plays a role in HR inhibition mediated by Tax warrants additional studies. Alternatively, it is possible that Tax expression prevents the recruitment of Rad51 to DDSB.

Our study describes the finding of a novel mechanism for retrovirus-associated genome instability. On one hand, HTLV-I Tax promotes DDSB during DNA replication and simultaneously prevents the repair machinery from using the conservative HR repair, thereby stimulating accumulation of genetic mutations and deletions. Surprisingly, HTLV-I also encodes another viral protein that inhibits HR repair and favors NHEJ. Abuse of unfaithful DNA repair through NHEJ in Tax-expressing cells is likely to have oncogenic consequences. Exactly how HR is inhibited and whether NHEJ is used as a default repair mechanism or is actively stimulated by Tax remain to be investigated. Our findings reveal an intriguing mechanism that could be involved in retrovirus-mediated cellular transformation and could explain the low incidence of HTLV-I-associated diseases, usually detected in 1 to 2% of infected individuals, and the very long latency, decades, before onset of the disease.
